# The CAT-SIR is out of the bag: tumors prefer host rather than dietary nutrients

**DOI:** 10.1186/s12915-021-01027-y

**Published:** 2021-05-10

**Authors:** Keene L. Abbott, Matthew G. Vander Heiden

**Affiliations:** 1 Koch Institute for Integrative Cancer Research and the Department of Biology, Massachusetts Institute of Technology, Cambridge, MA 02139 USA; 2 Dana-Farber Cancer Institute, Boston, MA 02215 USA

## Abstract

The extent to which tumors acquire nutrients from dietary sources as opposed to from the breakdown of host tissues is not known. In this issue of BMC Biology, Holland et al. report an approach where food sources with different isotope labeled carbon ratios can be used to answer this question, and find that tumors arising in *Drosophila melanogaster* procure most of their nutrients from the host.

## Commentary

Cancer cells must acquire nutrients from their environment in order to double biomass, turn over macromolecules, and maintain homeostasis. Thus, to meet the metabolic requirements of rapid proliferation, cancer cells display enhanced nutrient uptake compared to most non-transformed cells [1]. This is exemplified by elevated tumor glucose uptake, a property that is visualized in patients by FDG-PET scan [1]. Tracing the fate of isotope labeled glucose carbon (^13^C-glucose) in both animal models and patients has similarly revealed glucose uptake and metabolism by some cancers [1]. Isotope tracing and imaging studies have also shown cancers can utilize other nutrients, including material derived from autophagy to catabolize existing biomass [2]. However, whether the majority of nutrients used by specific cancers are sourced from the breakdown of pre-existing host stores or from dietary sources has been difficult to study with existing techniques.

### A method to distinguish diet versus host tissue nutrient acquisition

In this issue of BMC Biology, Holland et al. report a study where they leveraged the fact that plants with different photosynthetic carbon fixation pathways contain different ratios of ^13^C/^12^C carbon in biomass [3], which can be quantified using isotope ratio mass spectrometry [4]. So-called C4 plants that use a carbon fixation pathway involving 4-carbon molecules have a higher ^13^C/^12^C ratio than so-called C3 plants; therefore, diets derived from C3 or C4 plants have different ^13^C/^12^C ratios. To label the metabolites within the tissues of flies with ^13^C/^12^C ratios approximating that found in C3 or C4 plants, *D. melanogaster* eggs were laid on food sources derived from either C3 or C4 plants and the hatched larvae exhibited either a C3-type or C4-type ^13^C/^12^C ratio [3]. The authors used a well-established model of tumor induction in *D. melanogaster* driven by oncogenic Ras^V12^ expression and loss of the tumor suppressor gene *scribble* (*scrib*) [5], in which tumors form in the cephalic region of flies that can be separated from the rest of the host tissues. After allowing Ras^V12^, *scrib*^−/−^ *D. melanogaster* larvae to develop on the C3- or C4-type diets, the authors observed that the labeling was stable in host tissues over time. However, when the labeling was quantified in tumors, the ^13^C enrichment was found to steadily increase over time, achieving a ratio that was higher than that found even in C4 plants. This observation is not fully understood, but a previous study also found that tumor tissue had greater ^13^C enrichment compared to normal tissue, which was attributed to differences in the metabolism of transformed versus normal cells [6].

**Fig. 1:**
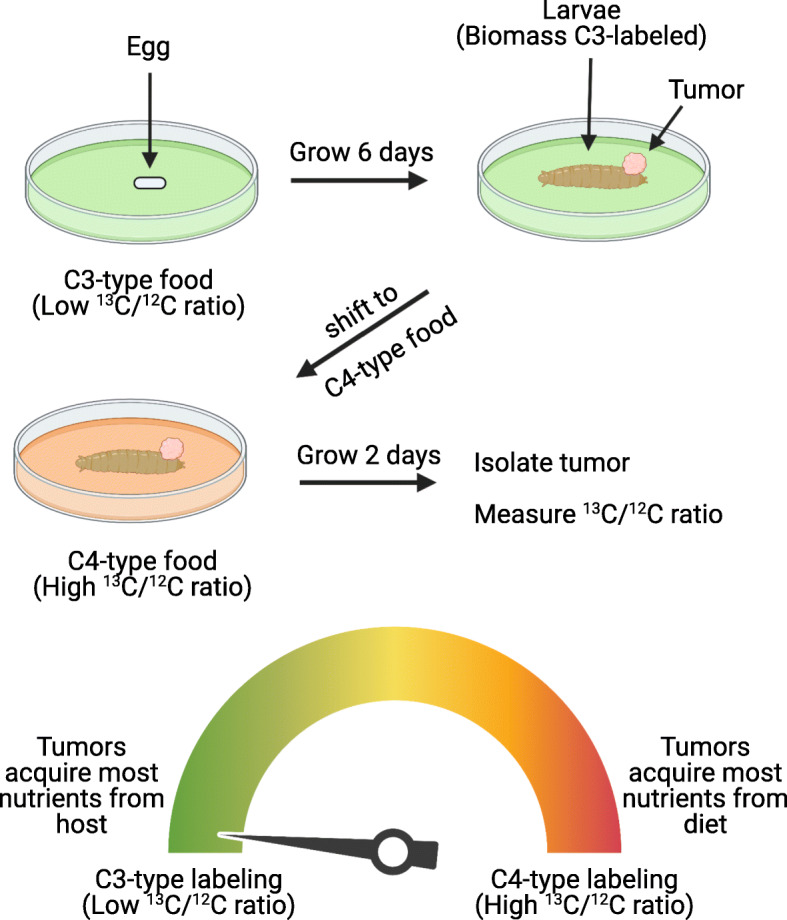
CATSIR is a method to determine whether tumors acquire nutrients from the host versus from the diet. Holland et al. took advantage of the fact that biomass from C3 and C4 plants have different ratios of ^13^C and ^12^C carbon. In their study, Ras^V12^, *scrib*^−/−^ *D. melanogaster* larvae were reared on a nutrient source derived from C3 plants for 6 days, which results in the larvae biomass having a ^13^C/^12^C ratio that approximates that of C3 plants. The larvae were then shifted to a nutrient source derived from C4 plants with a different ^13^C/^12^C ratio prior to isolating tumors. Thus, the ratio of ^13^C/^12^C ratio in the tumors reports the extent to which tumor nutrients were derived from host tissues versus from the diet

By taking advantage of the differential ^13^C/^12^C labeling ratios observed in flies reared on C3 versus C4 plant-derived food, the authors devised an experimental approach to determine the extent to which tumors derive their nutrients from the host or ingested food termed Carbon Transfer measured by Stable Isotope Ratios (CATSIR) (Fig. 1). For this approach, 6-day-old larvae are grown on C3-type food and then the food source is switched to a C4-type food for an additional 2 days. This resulted in a shift in the ^13^C/^12^C ratio of fly tissues, and the extent of this shift, relative to the starting ^13^C/^12^C ratio, can be used to infer how much carbon biomass in the tissue is derived from existing host biomass sources or from the diet. By using this approach in flies engineered to develop Ras^V12^, *scrib*^−/−^ tumors, the authors concluded that the tumors obtain the majority of their nutrients from the host, rather than from the diet. Importantly, the authors obtained the same results by performing the reverse experiment, by growing larvae on C4-type food and shifting to C3-type food, demonstrating the robustness of this methodology.

### Manipulating diet affects tumor nutrient uptake

Interestingly, the authors further noted that modifying the carbon source in the diet alters the balance of how nutrients are acquired by tumors in their model. For example, when the larvae were shifted to C4-type food containing mostly sugars, and lacking yeast as a source of lipids and amino acids, tumors acquired roughly the same amount of carbon from the diet as did larvae that were shifted to complete C4-type food. However, when larvae were shifted to C4-type food lacking sugar, tumors relied even more on host carbon sources. These data indicate that tumors will acquire dietary sugars when available, which is consistent with the fact that many tumors are glucose-avid [1]. Additionally, starving larvae by shifting them to food sources without nutrients caused tumors to rely completely on acquiring their nutrients from host biomass as might be expected. Of note, tumor growth rate was similar regardless of the diet used, even when larvae were starved, demonstrating the adaptability of tumors forming in this model to derive nutrients from the diet or from the host in different conditions. Exactly how altering diet affects nutrient acquisition by tumors is unclear and warrants further investigation.

### Future directions

The extent to which various tumors arising in mouse models also rely on host tissues as a major source of nutrients could be assessed by applying this methodology to mice fed C3- and C4-type diets. Feeding mice isotope-labeled diets can result in extensive biomass labeling in tissues [7], and mass spectrometry can be used to assess tissue biomass labeling [8]. A strength of the CATSIR approach is that it avoids a requirement for expensive isotope enriched material, and a need to assess labeling in individual biomass components. This relative simplicity could enable CATSIR to be used to study many different models in order to assess whether different genetic drivers or tumor sites affect the extent to which tumors acquire nutrients from dietary versus host sources. The approach is also amenable to studying how diet composition might lead to shifts in host nutrient utilization, as Holland et al. reported in their study. However, unlike *Drosophila* tumors, tumors that develop in mammals contain many non-cancer cell types that in some cases can be a major contributor to overall tumor mass [8], such that isotope ratios may need to be assessed in sorted cell populations [8].

The demonstration that some tumors can acquire most of their nutrients from the host leads to the question of how biomass is mobilized to feed the growing tumor. Some of the proposed mechanisms include phagocytosis, macropinocytosis [9], or additional means by which cancer cells drive release of nutrients from neighboring cells such as through autophagy [10]. This is a difficult question to tackle, as treating an organism with inhibitors of different uptake pathways can also alter whole-body metabolism, and the ability to genetically target specific tumor uptake pathways has been challenging. Nevertheless, CATSIR may prove useful as a low cost, relatively simple means to quantify the relative use of nutrients derived from host tissues, and these same strengths also argue for its application to that of non-cancer contexts, where differential utilization of host versus dietary nutrients may also play a role.

## Data Availability

Not applicable.

## References

[1] Faubert B, Solmonson A, DeBerardinis RJ. Metabolic reprogramming and cancer progression. Science. 2020;368(6487):eaaw5473. 10.1126/science.aaw5473. 10.1126/science.aaw5473 PMC7227780 32273439

[2] Poillet-Perez L, Xie X, Zhan L, Yang Y, Sharp DW, Hu ZS, Su X, Maganti A, Jiang C, Lu W, Zheng H, Bosenberg MW, Mehnert JM, Guo JY, Lattime E, Rabinowitz JD, White E (2018). Autophagy maintains tumour growth through circulating arginine. Nature..

[3] Holland P, Hagopian WM, Jahren AH, Rusten TE. Natural abundance isotope ratios to differentiate sources of carbon used during tumor growth in vivo. BMC Biol. 2021;19:85. 10.1186/s12915-021-01012-5. 10.1186/s12915-021-01012-5 PMC8108461 33966633

[4] Chartrand MMG, Mester Z (2019). Carbon isotope measurements of foods containing sugar: a survey. Food Chem.

[5] Brumby AM, Richardson HE (2003). Scribble mutants cooperate with oncogenic ras or notch to cause neoplastic overgrowth in drosophila. EMBO J.

[6] Tea I, Martineau E, Antheaume I, Lalande J, Mauve C, Gilard F, Barillé-Nion S, Blackburn AC, Tcherkez G (2016). 13C and 15N natural isotope abundance reflects breast cancer cell metabolism. Sci Rep.

[7] Mayers JR, Wu C, Clish CB, Kraft P, Torrence ME, Fiske BP, Yuan C, Bao Y, Townsend MK, Tworoger SS, Davidson SM, Papagiannakopoulos T, Yang A, Dayton TL, Ogino S, Stampfer MJ, Giovannucci EL, Qian ZR, Rubinson DA, Ma J, Sesso HD, Gaziano JM, Cochrane BB, Liu S, Wactawski-Wende J, Manson JAE, Pollak MN, Kimmelman AC, Souza A, Pierce K, Wang TJ, Gerszten RE, Fuchs CS, Vander Heiden MG, Wolpin BM (2014). Elevation of circulating branched-chain amino acids is an early event in human pancreatic adenocarcinoma development. Nat Med.

[8] Lau AN, Li Z, Danai LV, Westermark AM, Darnell AM, Ferreira R (2020). Dissecting cell-type-specific metabolism in pancreatic ductal adenocarcinoma. Elife..

[9] Recouvreux MV, Commisso C (2017). Macropinocytosis: a metabolic adaptation to nutrient stress in cancer. Front Endocrinol (Lausanne).

[10] Kimmelman AC, White E (2017). Autophagy and tumor metabolism. Cell Metab.

